# Oxford Nanopore Technology and its Application in Liquid Biopsies

**DOI:** 10.2174/0113892029286632231127055733

**Published:** 2023-12-28

**Authors:** Mariya Levkova, Trifon Chervenkov, Lyudmila Angelova, Deyan Dzenkov

**Affiliations:** 1 Department of Medical Genetics, Medical University Varna, Marin Drinov Str 55, Varna, 9000, Bulgaria;; 2 Laboratory of Medical Genetics, St. Marina Hospital, Hristo Smirnenski Blv 1, Varna, 9000, Bulgaria;; 3 Laboratory of Clinical immunology, St. Marina Hospital, Hristo Smirnenski Blv 1, Varna, 9000, Bulgaria;; 4 Department of General and Clinical Pathology, Forensic Medicine and Deontology, Division of General and Clinical Pathology, Medical University Varna, Marin Drinov Str 55, Varna, 9000, Bulgaria

**Keywords:** Nanopore, cancer, liquid biopsy, sequencing, cfDNA, cirDNA

## Abstract

Advanced medical technologies are transforming the future of healthcare, in particular, the screening and detection of molecular-genetic changes in patients suspected of having a neoplasm. They are based on the assumption that neoplasms release small amounts  of  various  neoplasm-specific molecules, such as tumor DNA, called circulating DNA (cirDNA), into the extracellular space and subsequently into the blood. The detection of tumor-specific molecules and specific molecular changes in body fluids in a noninvasive or minimally invasive approach is known as “liquid biopsy.” The aim of this review is to summarize the current knowledge of the application of ONT for analyzing circulating DNA in the field of liquid biopsies among cancer patients. Databases were searched using the keywords “nanopore” and “liquid biopsy” and by applying strict inclusion criteria. This technique can be used for the detection of neoplastic disease, including metastases, guiding precision therapy, and monitoring its effects. There are many challenges, however, for the successful implementation of this technology into the clinical practice. The first one is the low amount of tumor-specific molecules in the body fluids. Secondly, a tumor molecular signature should be discriminated from benign conditions like clonal hematopoiesis of unknown significance. Oxford Nanopore Technology (ONT) is a third-generation sequencing technology that seems particularly promising to complete these tasks. It offers rapid sequencing thanks to its ability to detect changes in the density of the electric current passing through nanopores. Even though ONT still needs validation technology, it is a promising approach for early diagnosis, therapy guidance, and monitoring of different neoplasms based on analyzing the cirDNA.

## 1 INTRODUCTION

Data on genetic alterations and molecular biomarkers that correlate to tumor growth and progression are accumulating [1]. New opportunities have emerged as a result of this for the detection and treatment of many cancers [1]. Along with other techniques employed in clinical oncology, pathology, and genetics, it also leads to the deployment of sensitive approaches for molecular-genetic alteration screening and detection.

Small amounts of cell-free circulating DNA are released from the tumors and identified in the bloodstream. These molecules have a short half-life, and within a few hours, the blood cell-free circulating tumor DNA would be completely degraded. It could, however, be isolated and examined using the so-called liquid biopsy technique [2]. This technology offers a non-invasive way to find tumors or monitor them, and it is highly promising. This could be explained by the fact that the amount of circulating tumor DNA is expected to correlate with the size of the tumor and its stage of development. It is possible to isolate genetic material from tumors that are challenging to access by traditional biopsy using liquid biopsies (LBs) [2].

**Fig. (1):**
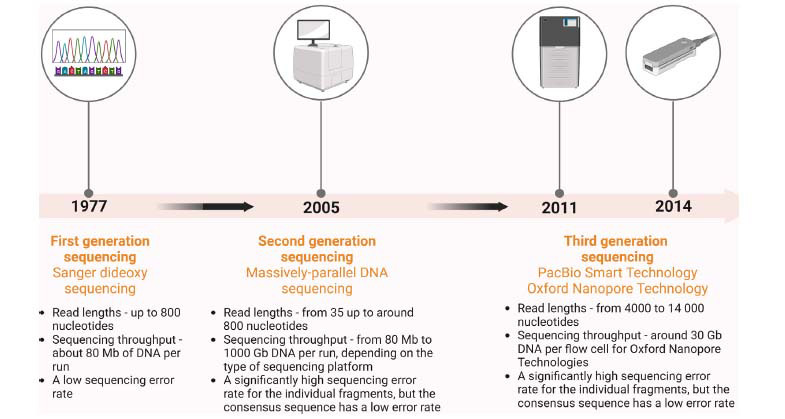
Evolution of the molecular-genetic technologies, used for sequencing. (Created in Biorender [44]).

The advancement in molecular-genetic analyses made it possible to identify the mutations typical for certain types of cancer, which could also be used for screening methods, such as collecting tumor DNA by using liquid biopsies. Sanger sequencing is first-generation sequencing, which was developed by Frederick Sanger in 1977. This method was later superseded by massively parallel sequencing, also called next-generation or second-generation sequencing, introduced in 2005 [3]. The third-generation sequencing debuted after 2010 and is also described as long-read sequencing. There are two technologies belonging to this generation – Pacific Biosciences (PacBio) and Oxford Nanopore technology (ONT) [3]. ONT can read a single DNA or RNA molecule, including circulating DNA (cirDNA), in real-time thanks to its nanopore sequencing, offering a short sequencing time (Fig. **1**). It is currently used in numerous research projects, including liquid biopsy, and shows promising results [3]. However, it has not been validated for single use in clinical practice.

The aim of this review is to provide an overview of the state of the art regarding Oxford Nanopore Technology and its use, advantages and disadvantages regarding its application in circulating tumor DNA analysis and liquid biopsies for different types of cancer.

## 2 STRUCTURE OF OXFORD NANOPORE TECHNOLOGY DEVICE

**Fig. (2):**
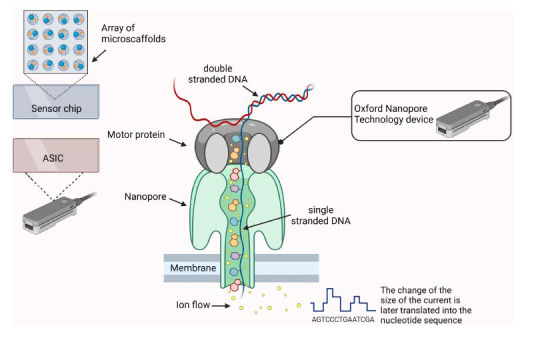
Structure of the Oxford Nanopore. The nanopores are located into an electro-resistant membrane, supported by an array of micro scaffolds. Each micro scaffold is connected by a corresponding electrode to a channel in the sensor array chip. Each nanopore channel is separately controlled and monitored by the Application-Specific Integrated Circuit (ASIC), located in the sensor chip of the nanopore device [4, 5]. (Created in Biorender).

Oxford Nanopore Technology is a third-generation sequencing technology that performs real-time sequencing. The DNA or RNA sequencing is done by adding samples to the so-called flow cells. Inside the flow cells are the nanopores, penetrating an electro-resistant membrane. This structure is strengthened by an array of micro scaffolds (Fig. **2**). Throughout the use of the nanopores, the array maintains its stability. Each micro scaffold has a corresponding electrode that is attached to a channel in the sensor array chip [4]. The flow cells have a different number of channels, depending on the device used. For example, one of the currently available, MinION, has 512 channels, which enables the real-time sequencing of up to 512 DNA or RNA molecules [5].

Each nanopore channel is separately controlled and monitored by the Application-Specific Integrated Circuit (ASIC). This makes it possible to conduct several nanopore runs simultaneously [5]. However, Lu *et al.* reported that some pores are more productive than others and can generate more data [5]. Also, some of the pores are not active at all during the run [5]. This is true also for the so-called wells. Four wells correspond to each channel and are connected to it. Before every run the four wells in every channel are tested in order to find the most active one. The latter is ranked as ‘g1’, the second most active is ‘g2’, *etc*. The collection of information will begin from the most active well, g1. This will be for the first half of the experiment - 24 hours. During the second half, data will be gathered from the other three wells [5].

## 3 PRINCIPLE OF NANOPORE SEQUENCING

When single-stranded DNA or RNA molecules pass through nanopores, the nucleic acid molecule and the nucleobases in particular, obstruct the pore and limit the electrical ionic current. As different nucleobases have different geometries, they obstruct the pore to a different extent, and thus, the change of electrical ionic current is sequence-dependent [6]. The change in electrical current in the nanopore is recorded by the ASIC and transferred to software, which uses a base-calling algorithm to infer the DNA or RNA sequence. This is performed by applying a machine learning approach, such as a hidden Markov model or recurrent neural network [6]. Ideally, the nanopore should accommodate a single nucleobase, but currently used nanopores accommodate several nucleobases. As a result, the electrical current depends on an oligonucleotide rather than a single nucleobase, which further complicates the base-calling process [7-9].

Importantly, each nucleobase(s) should reside in the nanopore for a minimum time needed for robust measurement, and this requires the use of dedicated mechanisms to impede and control the nucleic acid translocation through the pore. Currently, for this purpose, motor proteins –helicases are used. They cannot pass through the pore so their movement on single-stranded DNA or RNA molecule limits and determines the speed of translocation [9].

Helicases are preloaded on adapter oligonucleotides called “leader” or “hairpin,” which are ligated to the nucleic acid molecule during the process of library preparation for sequencing (Fig. **2**). The sequencing process will begin after the leader motor protein unzips the dsDNA, and the template strand could move through the pore. The hairpin motor protein, which is located at the end of the template strand, guides the translocation of the complement strand through the nanopore. After the translocation of the DNA through the nanopore is completed, the motor proteins are separated, and the whole process can be repeated until the sequencing is completed successfully [6].

As opposed to single-strand sequencing (called1D mode), when sequencing DNA, it is possible to sequence sequentially both complementary strands, which greatly improves the error rate: during analysis a consensus sequence that is generated by combining the base calls of opposite strands [6] is created. This is achieved by ligating a hairpin adapter on one end of DNA fragment (so-called 2D mode) or, in more recent protocols, ligating helicase-bearing adapters to both ends and increasing the probability that the complementary strand will immediately follow the first strand (so-called 1D2 mode) [9].

The above-described mechanism of sequencing provides real-time analysis, and the sequencing results are ready to be investigated right after the nucleotides have moved through the nanopore [9]. The specific structure of the ONT devices allows an increase in the read length, faster results and direct sequencing of the DNA or RNA with no need for prior PCR amplification [5]. Importantly, so-called rare bases are also identified because original nucleotides are called without preceding amplification [9]. Such bases like 5-methylcytosine (m5C) or 5-hydroxymethylcytosine (5hmC) in DNA are physical carriers of epigenetic information, so in addition to genetic information, epigenetic information is extracted in parallel [10, 11]. Similarly, epi transcriptomics (RNA epigenetics), which refers to the posttranscriptional modification of RNA bases (*i.e*., cytosine and adenosine methylation), information can be extracted when sequencing RNA [11].

## 4 TYPES OF ONT DEVICES

Oxford Nanopore Technologies introduced its first commercial sequencing device to the market, called the MinION, in 2014 [5]. Since then, the MinION has proven successful, probably due to its tiny size and affordable price [4]. After the launch of the MinION, there has been major progress in the number and characteristics of the available devices, responding to the professional needs of the end user. The different devices and their features are listed in Table **1**.

PromethION is available as P2 Solo, P2, PromethION 24, and PromethION 48. The difference is in the number of flow cells – the first two devices have two flow cells, the third one – 24, and the fourth one – 48 flow cells [12]. Due to the higher number of flow cells, PromethION offers a higher capacity to sequence bigger genomes, while MiniON cannot be used to sequence the whole human genome [12]. Oxford Nanopore Technologies launched the so-called Flongle. This is an adaptor, which allows for direct, real-time DNA or complementary DNA sequencing on smaller, single-use flow cells employing MinION or GridION. It is significantly cheaper compared to other devices – 90$ per flow cell, and generates up to 2.8 gigabytes data [12].

## 5 OXFORD NANOPORE TECHNOLOGY’S APPLICATION IN LIQUID BIOPSIES – ADVANTAGES AND DISADVANTAGES

The scientific community has been looking for a method of early cancer detection for an extensive period of time because an early diagnosis of cancer could result in a higher chance of survival. For example, between 60%–70% of patients who have pancreatic cancer present with inoperable tumor because it is locally advanced and/or there are metastases [13]. Delays in the diagnosis of breast cancer could also contribute to advanced-stage disease [14]. Therefore, a shorter period of time for treatment initiation could have a positive impact on the survival rate. However, traditional diagnostic techniques like computer tomography, magnetic resonance imaging, positron emission tomography, and histopathological analysis have different limitations. They cannot be used for an early screening approach due to their cost and potential harmful effects [15]. Liquid biopsies could transform cancer diagnostic and monitoring protocols as they could offer a minimally invasive and sensitive approach.

LBs are based on the analysis of tumor cells or their products, shed from the tumor and secreted in the biofluids, like, for example, cerebral spinal fluid, blood, *etc*. In most of the cases the preferred sample is peripheral blood [16]. The sample material may include extracellular vesicles, circulating RNA, plasma proteins and metabolites, all originating from the tumor cells, tumor-educated platelets, as well as circulating tumor cells or DNA. These biomolecules are released in the bloodstream of the patient as a result of the apoptosis, necrosis or phagocytosis of the tumor cells [17, 18]. According to a recently published guideline for the use of terms regarding cell-free DNA (cfDNA), the total amount of cfDNA in all types of body fluids can be named total cfDNA, whereas cfDNA, found in circulating body fluids (specifically blood and lymphatic fluid), is termed circulating DNA (cirDNA) [19] and this terminology is also used in the present review article.

The first publication, describing the presence of cirDNA in lupus patients, dates back to 1948 [20]. Later, it was established that there is also cirDNA from tumor cells and it was possible to use it as a tumor marker [21]. However, with the advancing genetic technologies, it became clear that cancer patients have larger levels of cirDNA than control persons, and this cirDNA is comprised of short fragments, mostly around 180 base pairs (bp) [22]. According to a number of studies, cirDNA fragments containing mutant alleles tend to be shorter than those containing wild-type alleles [23-25]. This is illustrated by a study focusing on a group of patients with lung cancer. The index patients had cirDNA that was approximately 134–144 bp longer and more fragmented than that of healthy individuals [23]. That is why monitoring the different sets of mutated variants and the fragment size of cirDNA may serve as a valuable screening approach [26]. Additionally, the genome of tumor cells and hence their cirDNA is noticeably different from that of normal cells - it exhibits an altered chromosomal number as well as other abnormalities, including point mutations and epigenetic alterations. These distinctive characteristics of tumor cirDNA can be identified with the help of sequencing technologies, such as ONT, making it possible to use cirDNA as a potential tumor biomarker for screening, treatment surveillance and prognosis [22]. Therefore, LBs have the potential to become a safer and reliable screening/diagnostic option, compared to conventional tissue biopsies. Because of its short lifespan, cirDNA derived from tumor cells could be used to monitor patients’ status and the therapeutic answer. However, this is also a disadvantage because it may cause difficulties isolating the tumor cirDNA and resulting in falsely negative findings [18]. The tumor cirDNA is also found in a low amount in the different body fluids and should be differentiated from benign conditions like clonal hematopoiesis of unknown significance [27]. This complication could be avoided by using a tumor molecular signature to distinguish them. The size of DNA fragments may also be utilized to differentiate them.

A significant development in the use of LBs was the FDA's approval in 2016 of the use of a commercially available test to detect structural alterations and pathogenic mutations in the *EGFR* gene in cirDNA of patients with non-small cell lung cancer as a companion diagnostic test [28]. Since then, there has been major progress in the application of LBs, particularly in the molecular genetic analyses used to search for pathogenic variants in the genome of cancer cells. In comparison to second-generation sequencing, third generation sequencing and ONT have been regarded as promising new techniques that might detect more information in a single test. With short sequencing times and quicker results, the new sequencing methods hold enormous potential for a thorough assessment of all tumor pathogenic variants and methylation status. The possible limitations, however, are high error rate, false positive and negative results and test cost [29].

There have been several published studies that used ONT to detect cirDNA in patients with lung cancer, prostate and ovarian cancer, head and neck squamous cell carcinoma, glioma, hepatocellular carcinoma, colorectal cancer, and different types of leukemia - B-cell acute lymphoblastic leukemia, chronic lymphocytic leukemia, and acute myeloid leukemia [2, 29-37]. In the majority of the investigations, the researchers collected a blood sample for the LBs. Interestingly, Sampathi *et al.* used bone aspirate to isolate the circulating tumor DNA, and Baslan *et al.* isolated it from cerebrospinal fluid [36, 37], which shows that the collected sample could vary depending on the characteristics of the tumor. Overall, the ONT was successful in detecting different pathogenic alterations in the circulating tumor DNA. For example, ONT found copy number variations and loss of heterozygosity when it was used as a screening approach for finding individuals with possible inherited breast cancer [33]. This demonstrates how LBs and ONT may be utilized as screening techniques for the early detection of cancer patients. In another study, ONT outperformed the Illumina platform because it discovered a single-exon inversion in *RAD51C* that targeted next-generation sequencing (NGS) would have missed and whose 5' breakpoint identification by short-read genome sequencing was unsuccessful [34]. Bruzek *et al.* reported 83% sensitivity and 100% specificity of the ONT after sequencing samples of pediatric high-grade glioma [35]. The high testing mobility and quick real-time sample processing of ONT were demonstrated by Euskirchen *et al.*, who used ONT to distinguish different types of central nervous system tumors. They designed a one-day ONT workflow, which allowed them to successfully identify copy number variants and methylation status of the tested tumor samples by drastically lowering the turn-around time [38].

However, all published articles recruited a small number of patients, which is a possible limitation of the studies. For example, Marcozzi *et al.* had only three test subjects with head and neck squamous cell carcinoma who were negative for a Herpes Virus infection [2]. The largest study was done by Dixon *et al.* They collected samples from 19 individuals who had a history of breast cancer in their families [33]. Another possible limitation is the lack of control participants in some of the studies [2, 29, 33, 35, 37]. Even though the molecular-genetic findings were confirmed using two types of analysis, ONT and sequencing, mostly on the Illumina platform, it is crucial to include control subjects in order to guarantee the accuracy of the collected information. However, validating the results by additionally sequencing the samples on Illumina or another platform, increases the cost of the LBs, which is important for the implementation into the clinical practice.

Moreover, Minervini *et al.* also discussed the significant error rate of ONT sequencing that could lead to a high proportion of false positive results. In order to avoid this limitation, their research group applied different error-correction tools by using different software programs - Nanocorrect correction pipeline and Amplicon Long-read Error Correction (ALEC) python script. They also used the mutation effect, allelic frequency and recurrence in order to filter the results and evade having false positive findings. With the help of the correction methods, they reported zero false negatives and a few false positive results [29].

An additional limitation of the ONT is the large amount of data which is generated during the sequence process – more than one terabyte. Due to the size of the data that must be uploaded to the cloud of the analyzing platform or the expensive computational infrastructure in order to store and analyze the sequencing data, there may be potential difficulties [39]. Another significant drawback is that some of the software used to analyze the results is open source and not validated. Oxford Nanopore launched EPI2ME solutions, but it requires the end user to upload the sequencing information to the cloud. All of this once more highlights the issue of upload speed, given the size of the generated data [40]. Also, the nanopores can be used for a limited number of experiments. Therefore, before each new run, specific wash kits should be used in order to clear the pores since these wash kits would digest nucleic acids from previous tests [4]. All of this would raise the price of LBs overall. At the moment, sequencing by ONT remains more expensive than using the most common sequencing platform – Illumina. By using Illumina, the expenses would range around 600$ for the whole human genome, while with the ONT, the costs would be around 1500$ [41, 42]. Last but not least, compared to short reads like Illumina, ONT has a significantly higher base calling error rate (1–10%) and poorer throughput, making analysis difficult [43]. This is why they should be used together with error correction tools, which could potentially complicate the analysis process.

## CONCLUSION

ONT offers a potential approach for screening, early diagnosis, and monitoring of various neoplasms. Despite its limitations, Nanopore sequencing has the potential to provide affordable genotyping, high testing mobility, and quick real-time sample processing. Therefore, it is considered the future of sequencing technologies and a promising strategy for the implementation of liquid biopsies into clinical practice when dealing with cancer patients.

## Figures and Tables

**Table 1:** Characteristics of the currently available Oxford Nanopore Devices.

**Type of ONT ** **Device**	**Number of Flow Cells**	**Number of ** **Channels / Flow Cell**	**Run Time**	**WGS - Small Genomes**	**WGS - Large ** **Genomes**	**Targeted ** **Sequencing**	**RNA ** **Sequencing**	**Epigenetics Analysis**	**Generated Data per Run**
MinION	1	512 channels,	72 hours	Yes	Yes, low-pass sequencing	Yes, recommended device	Yes	Yes	50 Gigabytes
GridION	1-5	512 channels	72 hours	Yes	Yes	Yes	Yes	Yes	250 Gigabytes
PromethION	1-48	2675 channels	72 hours	Yes	Yes, high coverage of large genomes	Yes	Yes	Yes	> 14 Terabytes, depending on the number of flow cells

**Note:** Adapted from https://nanoporetech.com/applications/dna-nanopore-sequencing.

## LIST OF ABBREVIATIONS

5hmC: 5-hydroxymethylcytosine
ALEC: Amplicon Long-read Error Correction
ASIC: Application-Specific Integrated Circuit
cfDNA: Cell Free DNA
cirDNA: circulating DNA
LBs: Liquid Biopsies
m5C: 5-methycytosine
NGS: Next-generation Sequencing
ONT: Oxford Nanopore Technology
PacBio: Pacific Biosciences
